# Oscillometric versus invasive blood pressure measurement in patients with shock: a prospective observational study in the emergency department

**DOI:** 10.1007/s10877-020-00482-2

**Published:** 2020-02-13

**Authors:** Agnes S. Meidert, Michael E. Dolch, Konstanze Mühlbauer, Bernhard Zwissler, Matthias Klein, Josef Briegel, Stephan Czerner

**Affiliations:** 1 Department of Anaesthesiology, University Hospital, LMU Munich, Marchioninistraße 15, 81377 Munich, Germany; 2 Emergency Department, Hospital of the LMU, Munich, Germany; 3 Department of Neurology, Hospital of the LMU, Munich, Germany; 4 Department of Anaesthesiology, Maria-Theresia-Klinik, Academic Teaching Hospital LMU Munich, Munich, Germany

**Keywords:** Emergency medicine, Oscillometric blood pressure, Shock, Resuscitation area, Hypotension

## Abstract

In emergency medicine, blood pressure is often measured by an oscillometric device using an upper arm cuff. However, measurement accuracy of this technique in patients suffering from hypotensive shock has not been sufficiently evaluated. We designed a prospective observational study investigating the accuracy of an oscillometric device in hypotensive patients admitted to the resuscitation area of the emergency department. Patients admitted to the resuscitation area of a university hospital, who were equipped with an arterial catheter and found to be hypotensive (mean arterial pressure (MAP) < 60 mmHg) were eligible for the study. Blood pressure was measured simultaneously via upper arm cuff and invasively under routine clinical conditions. After data extraction, Bland–Altman analysis, correlation coefficient and percentage error of mean and systolic blood pressure pairs were performed. We analysed 75 simultaneously obtained blood pressure measurements of 30 patients in hypotension, 11 (37%) were female, median age was 76.5 years (IQR 63–82). Oscillometric MAP was markedly higher than invasive MAP with a mean of the differences of 13 ± 15 mmHg (oscillometric—invasive), 95% limits of agreement − 16 to 41 mmHg, percentage error was 76%. In 64% of readings, values obtained by the upper arm cuff were not able to detect hypotension. Oscillometric blood pressure measurement is not able to reliably detect hypotension in emergency patients. Therefore, direct measurement of blood pressure should be established as soon as possible in patients suffering from shock.

## Purpose

Shock is defined as a “life threatening, generalised form of acute circulatory failure associated with inadequate oxygen utilisation by the cells” [1]. The presence of shock requires fast therapeutic intervention to prevent the patient from subsequent organ failure and death. Shock usually includes but is not limited to the presence of hypotension [1]. In order to detect hypotension in a patient, accurate and reliable blood pressure measurement is of utmost importance and guides the treatment of shock.

The reference method for arterial blood pressure in critical conditions is the direct measurement by an arterial catheter. However, usually only critically ill patients in the resuscitation area or on intensive care unit (ICU) are equipped with an arterial catheter. In emergency medicine, both in and out of the hospital, a patient’s blood pressure is measured noninvasively via an upper arm cuff. The noninvasive determination of blood pressure can be performed either manually or automatically. Automatic devices nowadays are commonly based on an oscillometric algorithm embedded in the device. Big database analyses showed that oscillometric techniques fail to reliably reflect very low or very high blood pressure values by overestimating low values and underestimating high values [2]. However, data comparing oscillometric with intra-arterial pressure measurements in hypotensive emergency patients with shock are sparse. Therefore, we designed a prospective observational study investigating the accuracy of an oscillometric device in hypotensive patients admitted to the resuscitation area of the emergency department.

## Methods

**Fig. 1:**
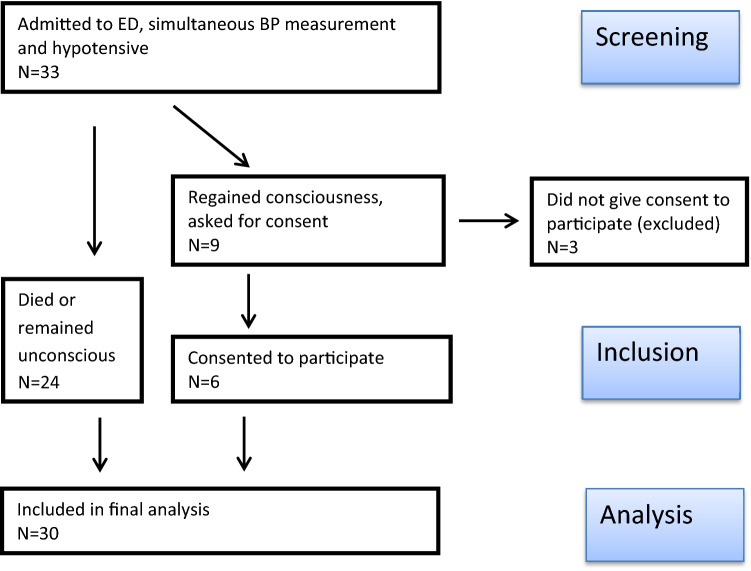
Flow chart of patient inclusion in the study

The aim of this prospective observational study was to investigate the accuracy of noninvasive oscillometric blood pressure measurement in hypotensive patients admitted to the emergency department. The local ethics committee (Ethikkommission bei der LMU München, Chairman W. Eisenmenger) approved the study under the Protocol Number 751-15 on 8th February 2016. The need for written informed consent prior to study inclusion was waived due to the emergency setting and the purely observational character of the investigation. In the follow up, subjects who survived and regained consciousness by hospital discharge were asked to participate in the study and to give written informed consent. For the others, it was presumed that there was no objection to participation (also see Fig. 1). The study took place in the emergency department (ED) of a German 2000-bed university hospital between April and June 2017. The study was designed and reported according to the STROBE guidelines [3]. Eligible for study inclusion were hypotensive patients admitted to the resuscitation area of the ED, where they were equipped with an arterial catheter. Exclusion criteria were the absence of spontaneous circulation and rupture or occlusion of central arteries. Hypotension was defined as intra-arterial mean arterial pressure below 60 mmHg. Since guidelines for treatment of septic shock suggest keeping mean arterial pressure at least at 65 mmHg [4], 60 mmHg or less can be regarded as severe hypotension in patients treated in the resuscitation area. Large cohort analyses revealed that a MAP < 60 mmHg was associated with increased risk of organ injuries such as myocardial infarction and acute kidney failure [5, 6]. Therefore, the institutional “hypotension threshold” is 60 mmHg for adult patients. We chose primarily to analyse mean arterial pressure as it is not as affected by the measurement site of the arterial catheter as systolic arterial pressure [7]. In addition, we analysed the accuracy of systolic arterial pressure because low systolic blood pressure is—amongst others—one sign in early warning scores, e.g. used by rapid response teams [8].

In the resuscitation area, patients’ blood pressure was monitored noninvasively by an oscillometric upper arm cuff (Dräger Infinity® M540; Dräger, Lübeck, Germany) in the correct size (either M, M+ or L) and, as soon as possible invasively via arterial cannulation of the radial, brachial or femoral artery, according to the decision of the anaesthesiologist in charge. Noninvasive blood pressure measurement was set up by a trained nurse, who also checked correct zeroing and flushing of the invasive blood pressure measurement as well as the correct position of the pressure transducer at level of the right atrium. In case of cannulation of the radial or brachial artery, the nurse ensured that the upper arm cuff was placed at the contralateral arm. The interval for automatic oscillometric measurements was standardly set at 3 min. All measurements were performed in a supine position. No study-specific intervention took place. All vital parameters of the patients including blood pressure values were stored in the bedside computer. When direct blood pressure measurement was established, the simultaneously recorded blood pressure values were extracted from the bedside computer, screened for hypotensive intra-arterial values, checked for plausibility (i.e. difference between invasive systolic and diastolic blood pressure < 7 mmHg, systolic blood pressure < 40 mmHg, mean arterial pressure < 30 mmHg) and artefacts (e.g., damping phenomena, static pressure during blood withdrawal, etc.) according to a predefined protocol, and analysed. All plausible values were included as long as the patient remained hypotensive, leading to a different number of measurements per patient (e.g. in case of refractory hypotension). The patient monitors in the resuscitation areas were checked for calibration and other technical errors by qualified technicians according to German regulations.

Patients’ demographic data were extracted from the medical records and the patient data management system (NarkoData; Imeso, Gießen, Germany), as were patients’ arterial lactate level, pH and base excess.

### Statistical analysis

A previous study comparing oscillometric and invasive blood pressure found a mean difference of 8 mmHg at the hypotension threshold of 60 mmHg mean arterial pressure [2]. The standard deviation (SD) in this blood pressure range is 11 mmHg. Based on these values, a confidence level of 95% and a power of 80%, the recommended sample size was 30.

For data analysis, we used the software Excel (Microsoft, Redmond, US) and R (The R project for statistical computing, Vienna, Austria). Simultaneously obtained mean and systolic arterial blood pressure values of each patient were analysed by the Bland–Altman method accounting for repeated measurements, resulting in bias (± SD) and 95% limits of agreement (bias ± SD × 1.96) [9]. An error grid analysis for comparison of arterial pressure method comparison studies was performed [10]. This analysis classifies the difference between the test method (oscillometric blood pressure) and the standard method (invasive blood pressure) in risk categories based on the aggregated opinion of 25 experts. A difference in blood pressure can either be clinically irrelevant, for example if the oscillometric cuff measures a mean arterial pressure of 100 mmHg while the invasive mean arterial pressure (standard method) is 80 mmHg, or in varying degrees dangerous for the patient (e.g. oscillometry measures mean arterial pressure of 70 mmHg, invasive mean arterial pressure is 50 mmHg). The difference between methods is 20 mmHg in both cases, although the former is irrelevant, the latter potentially dangerous for the patient. The error grid analysis allows a classification of measurement differences depending on their clinical relevance (regarding unnecessary treatment or no treatment, although indicated), ranging from A (no risk, no difference in clinical action), B (low risk, benign or no treatment), C (moderate risk, unnecessary treatment with moderate non-life-threatening consequences), D (significant risk, unnecessary treatment with severe non-life-threatening consequences) to E (high risk, unnecessary treatment with life-threatening consequences) for the patient [10]. Error grid for mean arterial pressure and systolic arterial pressure and the proportion of measurements in the different risk categories were calculated using the software by Grothe et al. [11] Pearson’s correlation coefficient and percentage error [12] were calculated. Blood pressure values were reported as mean ± SD and range.

Subgroup analyses were not performed due to the small number of patients included.

Patients’ characteristics were calculated as absolute and relative frequencies, and median and interquartile range (not normally distributed), as were laboratory values from blood gas analysis.

To summarise the endpoint, we aimed to investigate the accuracy of noninvasive intermittent blood pressure measurement via an oscillometric upper arm cuff in 30 patients suffering from shock treated in the resuscitation area in a clinical method comparison study using invasive blood pressure measurement as the reference method.

## Results

Within the study period 33 patients with simultaneous invasive and noninvasive blood pressure measurement who had a mean arterial pressure below 60 mmHg were admitted to the emergency department. Figure 1 shows a flow chart of patient inclusion. 30 patients were included in the final analysis. Body mass index was available for 27 patients. Results of blood gas analysis and lactate levels were available for 29 patients. Table 1 shows patients’ characteristics.

**Table 1:** Patients’ characteristics

Age, y	76.5	(63–82)
Female, n	11	(37%)
Body mass index	25	(23–26)
Reason for admission		
Cardiocirculatory failure, n	8	(27%)
Sepsis, n	8	(27%)
Trauma, n	5	(17%)
Thrombembolism, n	4	(13%)
Respiratory failure, n	3	(10%)
Other, n	2	(7%)
Laboratory results		
Lactate (mmol/L)	3.3	(1.4–9.0)
Base excess	− 5.4	(− 10.3 to − 2.8)
pH	7.308	(7.164–7.388)

Values are presented either as n (%) or median (IQR); percentages may not sum up to 100 due to rounding

Invasive blood pressure was measured in 19 (63%) cases in the radial artery, in 9 (30%) in the femoral and in 2 (7%) in the brachial artery.

**Fig. 2:**
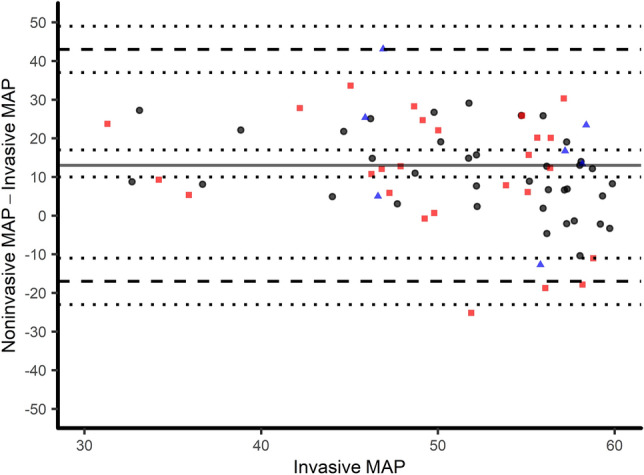
Modified Bland–Altman-Plot of oscillometric and invasive mean arterial pressure in mmHg. Invasive pressure values on the x-axis are plotted against the bias (oscillometric—invasive) of each pair on the y-axis. The bold line corresponds to the mean difference, dashed lines show 95% limits of agreement, the dotted lines indicate the corresponding 95% confidence intervals. Radial, brachial and femoral measurements are black, blue and red, respectively

**Fig. 3:**
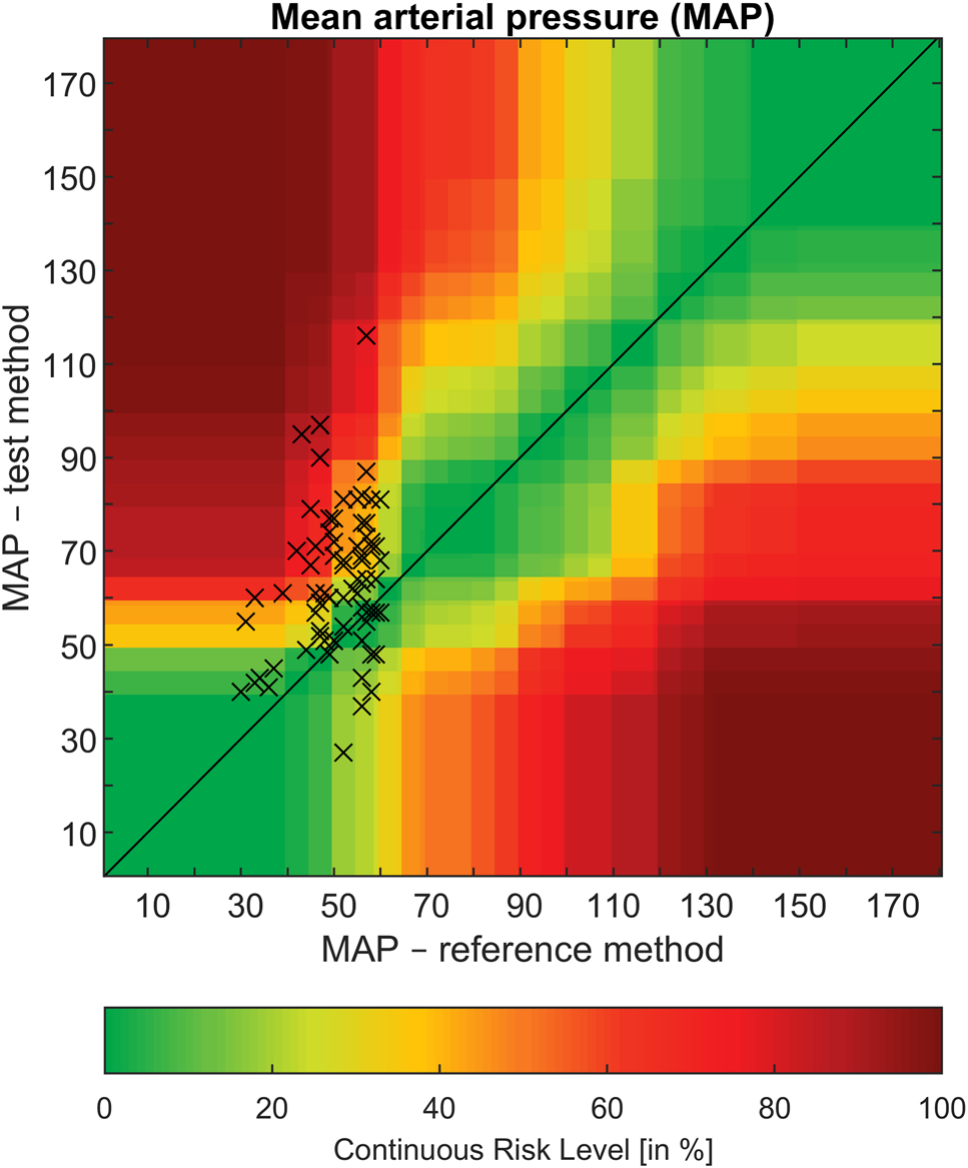
Error grid for mean arterial pressure in mmHg. Invasive pressure (x-axis, reference method) is plotted against noninvasive pressure (y-axis, test method), the colours indicate the continuous risk level from green (zone A, no risk) to dark red (zone E, dangerous risk), based on experts’ clinical judgement [10]

Seventy-seven simultaneous invasive and noninvasive measurements were recorded during hypotension. After screening for artefacts and excluding these values, 75 data sets of blood pressure measurement were analyzed. The number of measurements per patient ranged from 1 to 10 (median 1, IQR 1–3). Invasive mean arterial pressure was 51 ± 8 mmHg (30–60 mmHg), whereas noninvasively obtained mean arterial pressure was 64 ± 15 mmHg (27–116 mmHg). The correlation of invasive versus noninvasive mean arterial pressure values resulted in a correlation coefficient of 0.26 (p = 0.024) (Fig. 3). Oscillometric values were markedly higher than the invasively measured mean arterial pressure: Bland–Altman analysis revealed a mean of the differences of 13 ± 15 mmHg (oscillometric—invasive), resulting in 95% limits of agreement from − 16 to 41 mmHg (Fig. 2). The error grid analysis classified 23, 34, 32, 9 and 1% of measurements in the risk categories A–E, respectively. Based on this analysis, in 42% of paired measurements noninvasive blood pressure differed from the invasive blood pressure with high clinical relevance (moderate to dangerous risk for the patient, Fig. 3). 48 (64%) of noninvasive values measured a mean arterial pressure above 60 mmHg and therefore failed to meet our hypotension threshold at all.

**Fig. 4:**
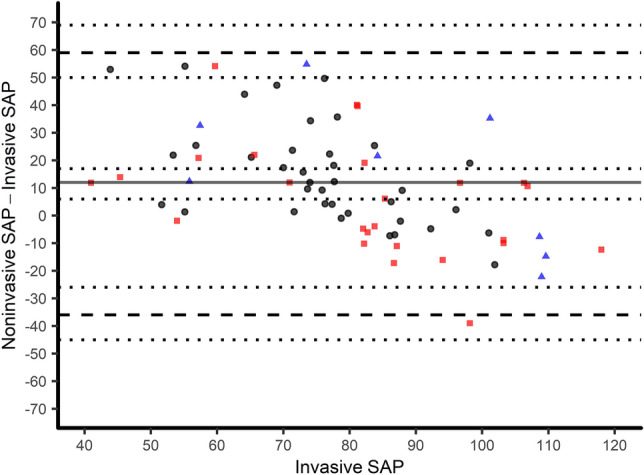
Modified Bland–Altman-Plot of oscillometric and invasive systolic arterial pressure in mmHg. Invasive pressure values on the x-axis are plotted against the bias (oscillometric—invasive) of each pair on the y-axis. The bold line corresponds to the mean difference, dashed lines show 95% limits of agreement, the dotted lines indicate the corresponding 95% confidence intervals. Radial, brachial and femoral measurements are black, blue and red, respectively

**Fig. 5:**
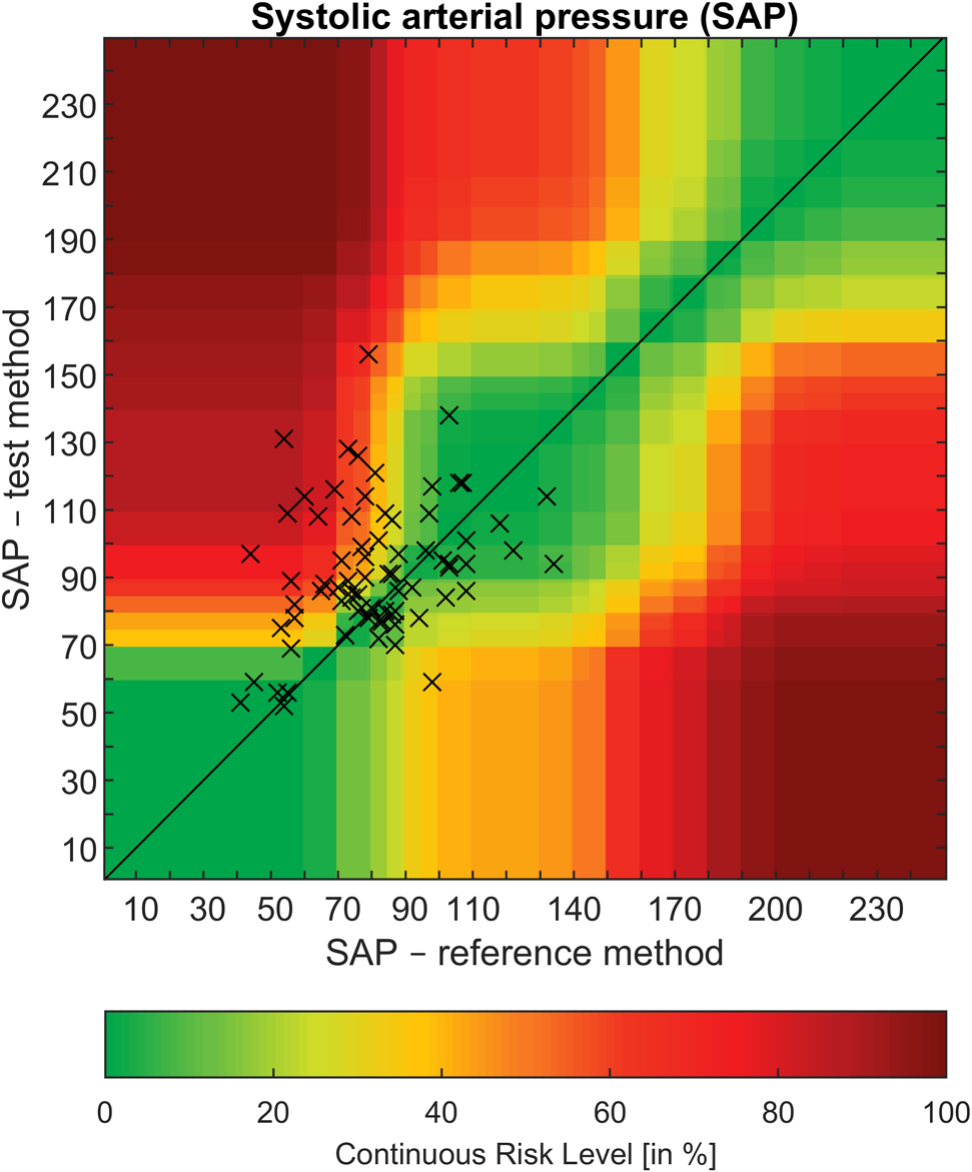
Error grid for systolic arterial pressure in mmHg. Invasive pressure (x-axis, reference method) is plotted against noninvasive pressure (y-axis, test method), the colours indicate the continuous risk level from green (zone A, no risk) to dark red (zone E, dangerous risk), based on experts’ clinical judgement [10]

Values for invasive systolic arterial pressure were 81 ± 20 mmHg and ranged from 41 to 134 mmHg, whereas noninvasive systolic arterial pressure was 93 ± 20 mmHg (52–156 mmHg). The correlation coefficient for systolic arterial pressure was 0.29 (p = 0.010) (Fig. 5). Analogue to the findings for mean arterial pressure, systolic pressure obtained by the oscillometric cuff was markedly higher: The mean of the differences was 12 ± 24 mmHg (oscillometric—invasive) with 95% limits of agreement ranging from − 36 to 59 mmHg (Fig. 4). The difference between systolic measurements analyzed with the error grid method showed clinically relevant deviation in 30% of measurements (class C–E; moderate to dangerous risk), with 50 and 20% of measurements in class A and B, respectively (Fig. 5). The percentage error was 76% and 63% for mean and systolic arterial pressure, respectively.

## Discussion

We aimed to investigate the reliability of blood pressure measurement using an oscillometric device in unstable emergency patients. Our data show that it is not possible to reliably detect hypotension in the majority of patients with shock using the tested noninvasive oscillometric device.

In our hypotensive patient cohort, oscillometric measurements provided values of mean arterial pressure that were on average 13 mmHg higher than values obtained by invasive blood pressure measurement, which is considered the reference standard. This finding is in accordance with other results investigating oscillometric determination of blood pressure retrospectively [2, 13]. Wax and colleagues analysed simultaneous blood pressure data of 15,310 patients perioperatively [2]. They described a relevant overestimation of hypotensive values by the oscillometric device [2]. However, the number of cases in the hypotensive range was relatively small. Similarly, a study by Lehman et al. demonstrated the same tendency with good agreement in the normotensive range of blood pressure values in critically ill patients with both oscillometric and invasive blood pressure measurement [13]. Recently, Seidlerová et al. studied 85 patients admitted for cardiogenic shock after stabilisation on intensive care unit. In accordance to our results, accuracy of noninvasive oscillometric measurement was affected by hypotension [14].

The reason for overestimation in hypotension may lie within the embedded measurement algorithm of oscillometric devices: They analyse oscillations of the vessel during compression, transmitted through the air-filled occluding cuff. Most commonly, mean arterial pressure is set at the greatest amplitude of oscillations whereas systolic and diastolic pressure are determined by the means of proprietary envelope curves applied to the individual course of oscillations during the measurement cycle. However, the algorithms were often developed in normotensive individuals and analyse oscillations with amplitudes of 1–5 mmHg at the most [15]. In patients with hypotension and shock, vasoconstriction and low output may impair the normal physiology of the vascular tree and therefore make reliable detection of blood pressure from oscillations in the vessel highly inaccurate. In our patient collective, sole oscillometric monitoring would have left hypotension below 60 mmHg undetected in 64% of measurements. Nonetheless, oscillometric devices are most commonly used for monitoring of blood pressure as well as guidance of vasopressor or fluid therapy in emergency medicine and perioperatively [16]. Even in intensive care units and the resuscitation area, oscillometric blood pressure measurement is considered a good alternative to the arterial line [17]. This is partially due to several advantages of oscillometric blood pressure determination over direct measurement, namely its fast establishment, ease of use and automated mode of monitoring. In addition, many clinicians may not know about the limitation in patients with shock, because data on the measurement performance in unstable patients are rare. Nevertheless, Lakhal et al. explicitly state in their review, that oscillometric measurement can safely rule out hypotension (mean arterial pressure below 65 mmHg) [16], which is not in accordance to our findings.

Timely and accurate detection of hypotension in the emergency department is of great importance for several reasons. First, algorithms for rapid response teams base the distinction between unstable or stable amongst other factors on presence of hypotension [8]. Second, hypotension is associated with impaired organ perfusion, especially of the brain, heart and kidney. Periods of low blood pressure can result in organ dysfunction or failure [6]. Furthermore, hypotension is part of many early warning scores such as the qSOFA score for those at risk of septic shock [18]. Therefore hypotension is seen as a surrogate of hypoperfusion and the resulting lack of oxygen delivery requires immediate treatment [4]. However, our data show that there are patients in which hypotension is likely to remain undetected. A survey by Chatterjee et al. among intensivists revealed, that 47% of physicians use noninvasive blood pressure measurement to guide vasopressor therapy [17]. The widespread use of oscillometric devices in these patients may lead to a significant undertreatment in a most serious condition. Especially young patients may compensate the lack of oxygen for some time while remaining “stable” in terms of not developing hypotension, a fact that may delay diagnosis of shock. Rather than only using noninvasive blood pressure values for guiding fluid and vasopressor therapy, we suggest that clinical and laboratory signs such as vigilance, mottling and temperature of the skin, capillary refill time, urine output and lactate level should be taken into account [19–21]. In addition, invasive blood pressure measurement should be established as soon as possible in patients with suspected shock.

There are limitations of our study. The study did not follow a strict protocol with respect to number of measurements per patient or site of arterial cannulation, because it was designed to catch realistic clinical conditions. Due to the emergency setting, there was no testing for a possible blood pressure difference between arms. Different arterial cannulation sites may introduce bias, however differences between radial and femoral mean arterial pressure are clinically not relevant [22]. Another possible limitation is, that the device of only one manufacturer was studied. So, we are not able to conclude on other oscillometric devices. The proprietary algorithms embedded in oscillometric devices are in most cases not publicly available for scientific evaluation, so it is possible that results for other oscillometric devices may differ. However, all algorithms are based on the oscillogram detected during a measurement cycle. A possible explanation for the observed inaccuracy in hypotension is a weaker signal of oscillations when arterial blood pressure is low. If a weaker signal is the reason for our findings, the performance of devices from other manufacturers in hypotension might be similar. Another possible explanation is that the algorithm used is being based predominantly on normotensive pressures. For severe hypotension linked to shock specific algorithms with higher accuracy are mandatory. To study the generalisability of our findings in hypotension for all oscillometric devices, a larger multicentre trial investigating different devices is needed.

The strength of our study is the prospective approach of an emergency patient cohort suffering from shock. Data of patients in profound shock are difficult to obtain as therapeutic efforts must be made immediately in this critical state. In addition, we studied all patients in the supine position and checked for adequate positioning of pressure transducer and cuff at the mid-chest level. Another strength is that we only analysed data pairs in true hypotensive state that were not affected by artefacts. Finally, invasive arterial measurements were used as reference method which is regarded as gold standard in other critical conditions such as cardiac surgery or critical care medicine.

In conclusion, oscillometric blood pressure measurement is not able to reliably detect hypotension in emergency patients. Therefore, direct measurement of blood pressure should be established as soon as possible to guide vasopressor and fluid therapy in patients suffering from shock. Larger clinical trials are needed to further investigate the impact of oscillometric and invasive blood pressure monitoring in emergency patients with shock on therapeutic decisions and patient’s outcome.
