# Correction to: Oscillometric versus invasive blood pressure measurement in patients with shock: a prospective observational study in the emergency department

**DOI:** 10.1007/s10877-021-00766-1

**Published:** 2021-10-15

**Authors:** Agnes S. Meidert, Michael E. Dolch, Konstanze Mühlbauer, Bernhard Zwissler, Matthias Klein, Josef Briegel, Stephan Czerner

**Affiliations:** 1 Department of Anaesthesiology, University Hospital, LMU Munich, Marchioninistraße 15, 81377 Munich, Germany; 2 Emergency Department, Hospital of the LMU, Munich, Germany; 3 Department of Neurology, Hospital of the LMU, Munich, Germany; 4 Department of Anaesthesiology, Maria‑Theresia‑Klinik, Academic Teaching Hospital LMU Munich, Munich, Germany

## Correction to: Journal of Clinical Monitoring and Computing (2021) 35:387–393 10.1007/s10877-020-00482-2

The article: “Oscillometric versus invasive blood pressure measurement in patients with shock: a prospective observational study in the emergency department”, written by Agnes S. Meidert, Michael E. Dolch, Konstanze Mühlbauer, Bernhard Zwissler, Matthias Klein, Josef Briegel and Stephan Czerner, was originally published electronically on the publisher’s internet portal on 13 February 2020 without open access. With the author(s)’ decision to opt for Open Choice the copyright of the article changed on 18 October 2021 to ©The Author(s) 2021 and the article is forthwith distributed under a Creative Commons Attribution 4.0 International License, which permits use, sharing, adaptation, distribution and reproduction in any medium or format, as long as you give appropriate credit to the original author(s) and the source, provide a link to the Creative Commons licence, and indicate if changes were made. The images or other third party material in this article are included in the article’s Creative Commons licence, unless indicated otherwise in a credit line to the material. If material is not included in the article’s Creative Commons licence and your intended use is not permitted by statutory regulation or exceeds the permitted use, you will need to obtain permission directly from the copyright holder. To view a copy of this licence, visit http://creativecommons.org/licenses/by/4.0/. The original article has been corrected.

